# Pathophysiology of cholestatic liver disease and its relevance for in vitro tests of hepatotoxicity

**DOI:** 10.17179/excli2016-864

**Published:** 2016-12-23

**Authors:** Regina Stöber

**Affiliations:** 1 Leibniz Research Centre for Working Environment and Human Factors at TU Dortmund (IfADo), Ardeystrasse 67, 44139 Dortmund, Germany

## ⁯⁯⁯

Recently, Peter Jansen from the Department of Gastroenterology and Hepatology and colleagues published a comprehensive review about the mechanisms leading to cholestatic liver disease (Jansen et al., 2016[6]). The authors describe the morphological changes at different topological domains of the biliary tree: while large bile ducts respond by enlargement of ductular diameter to maximize their volume, intralobular bile ducts respond by branching and surface corrugation to optimize their capacity to reabsorb bile salts (Vartak et al., 2016[13]). A key mechanism in cholestatic liver disease is that the bile canaliculi in liver lobules become leaky, and toxic bile can get into contact with parenchymal cells leading to cytotoxicity and necrosis, a phenomenon also named bile infarct.

For toxicologists the perhaps most important lesson learned from this review is that cholestatic liver disease may have an ascending and a descending pathophysiology. For example primary sclerosing cholangitis and primary biliary cholangitis begins with early lesions 'downstream' in bile ducts which leads to bile salt-mediated injury 'upstream' in liver parenchyma. In contrast, most forms of drug induced cholestasis have a descending pathophysiology, where damage of hepatocytes represents the initial key event.

Considering this classification it may be justified that in vitro systems to identify hepatotoxic compounds focus on hepatocytes (Miszczuk et al., 2015[7]; Tolosa et al., 2015[12]; Björnsson, 2015[2]; Stöber, 2015[10][11]). Currently, large research programs focus on hepatocyte in vitro systems, either using functional assays (Godoy et al., 2013[4]; Reif et al., 2016[8]) or genome wide expression analysis (Schaap et al., 2015[9]; Benet et al., 2014[1]; Grinberg et al., 2014[5]).

However, it should be taken into account that also toxic cholestasis may in rare cases have an ascending pathophysiology. For example, 3,5-diethoxycarbonyl-1,4-dihydrocollidine (DDC) primarily causes damage to bile ducts, while parenchymal damage occurs as a secondary event (Fickert et al., 2007[3]). 

Therefore, insufficient sensitivity in currently performed in vitro screens for hepatotoxicity may be a consequence of neglecting compounds acting by an ascending pathophysiology, where cholangiocytes and not hepatocytes represent primary targets.

## References

[1] Benet M, Moya M, Donato MT, Lahoz A, Hervás D, Guzmán C (2014). A simple transcriptomic signature able to predict drug-induced hepatic steatosis. Arch Toxicol.

[2] Björnsson ES (2015). Drug-induced liver injury: an overview over the most critical compounds. Arch Toxicol.

[3] Fickert P, Stöger U, Fuchsbichler A, Moustafa T, Marschall HU, Weiglein AH (2007). A new xenobiotic-induced mouse model of sclerosing cholangitis and biliary fibrosis. Am J Pathol.

[4] Godoy P, Hewitt NJ, Albrecht U, Andersen ME, Ansari N, Bhattacharya S (2013). Recent advances in 2D and 3D in vitro systems using primary hepatocytes, alternative hepatocyte sources and non-parenchymal liver cells and their use in investigating mechanisms of hepatotoxicity, cell signaling and ADME. Arch Toxicol.

[5] Grinberg M, Stöber RM, Edlund K, Rempel E, Godoy P, Reif R (2014). Toxicogenomics directory of chemically exposed human hepatocytes. Arch Toxicol.

[6] Jansen PL, Ghallab A, Vartak N, Reif R, Schaap FG, Hampe J (2016). The ascending pathophysiology of cholestatic liver disease. Hepatology.

[7] Miszczuk GS, Barosso IR, Zucchetti AE, Boaglio AC, Pellegrino JM, Sánchez Pozzi EJ (2015). Sandwich-cultured rat hepatocytes as an in vitro model to study canalicular transport alterations in cholestasis. Arch Toxicol.

[8] Reif R, Ghallab A, Beattie L, Günther G, Kuepfer L, Kaye PM (2016). In vivo imaging of systemic transport and elimination of xenobiotics and endogenous molecules in mice. Arch Toxicol.

[9] Schaap MM, Wackers PF, Zwart EP, Huijskens I, Jonker MJ, Hendriks G (2015). A novel toxicogenomics-based approach to categorize (non-)genotoxic carcinogens. Arch Toxicol.

[10] Stoeber R (2015). Drug-induced mitochondrial impairment in liver cells. EXCLI J.

[11] Stoeber R (2015). Transcriptomic signature for drug-induced steatosis. EXCLI J.

[12] Tolosa L, Gómez-Lechón MJ, Donato MT (2015). High-content screening technology for studying drug-induced hepatotoxicity in cell models. Arch Toxicol.

[13] Vartak N, Damle-Vartak A, Richter B, Dirsch O, Dahmen U, Hammad S (2016). Cholestasis-induced adaptive remodeling of interlobular bile ducts. Hepatology.

